# The diagnostic challenge of pneumocystis pneumonia and COVID‐19 co‐infection in HIV

**DOI:** 10.1002/rcr2.725

**Published:** 2021-02-22

**Authors:** Alistair G.B. Broadhurst, Usha Lalla, Jantjie J. Taljaard, Elizabeth H. Louw, Coenraad F.N. Koegelenberg, Brian W. Allwood

**Affiliations:** ^1^ Division of General Medicine, Department of Medicine, Faculty of Medicine and Health Sciences Stellenbosch University and Tygerberg Hospital Cape Town South Africa; ^2^ Division of Pulmonology, Department of Medicine, Faculty of Medicine and Health Sciences Stellenbosch University and Tygerberg Hospital Cape Town South Africa; ^3^ Division of Infectious Diseases, Department of Medicine, Faculty of Medicine and Health Sciences Stellenbosch University and Tygerberg Hospital Cape Town South Africa

**Keywords:** COVID‐19, HIV, pneumocystis pneumonia, SARS‐CoV‐2

## Abstract

Coronavirus disease 2019 (COVID‐19) and pneumocystis pneumonia (PCP) share many overlapping features and may be clinically indistinguishable on initial presentation in people living with HIV. We present the case of co‐infection with COVID‐19 and PCP in a patient with progressive respiratory failure admitted to our intensive care unit where the dominant disease was uncertain. This case highlights the difficulty in differentiating between the two diseases, especially in a high HIV prevalence setting where PCP is frequently diagnosed using case definitions and clinical experience due to limited access to bronchoscopy, appropriate laboratory testing, and computed tomography scans. In addition, diagnostic testing may yield false‐negative results in both diseases, and clinician awareness to the overlap and pitfalls is essential if COVID‐19 becomes endemic in such settings.

## Graphical abstract

We present the case of a 54‐year‐old male with newly diagnosed advanced HIV who presented with clinical, biochemical, and radiological features that were consistent with both pneumocystis pneumonia (PCP) and coronavirus disease 2019 (COVID‐19). He subsequently tested positive for both diseases; however, the contribution of each disease to his progressive respiratory failure is unclear.

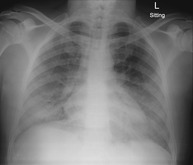

## Introduction

Local data suggest that people living with HIV (PLWH) and people infected with tuberculosis (TB) may have an increased risk of mortality with severe acute respiratory syndrome coronavirus 2 (SARS‐CoV‐2) co‐infection [1]. There are reports of SARS‐CoV‐2 co‐infection with other opportunistic infections in PLWH [2]. Pneumocystis pneumonia (PCP) has considerable clinical overlap with SARS‐CoV‐2 pneumonia, as both diseases may have a sub‐acute presentation, with dry cough and dyspnoea, and result in hypoxic pneumonia with bilateral ground‐glass infiltrates, lymphopaenia, and an elevated lactate dehydrogenase (LDH) [3, 4]. We present a patient admitted with concomitant PCP and SARS‐CoV‐2 infection.

## Case Report

A 54‐year‐old man was admitted to a district hospital, with a three‐week history of cough, myalgia, fever, and progressive dyspnoea. His body mass index was within the normal range, and he was known with an eight‐year history of hypertension and type 2 diabetes with evidence of left ventricular hypertrophy on electrocardiogram. He reported two previous episodes of drug‐sensitive pulmonary TB for which he completed treatment.

On admission, he tested HIV positive and his CD_4_ count was 26 cells/μL, with a viral load of 2,447,646 copies/mL. His nasopharyngeal swab for SARS‐CoV‐2 polymerase chain reaction (PCR) was also positive. He was transferred to a coronavirus disease (COVID) field hospital requiring nasal prong oxygen to maintain an oxygen saturation (SpO_2_) of 96%.

**Figure 1:**
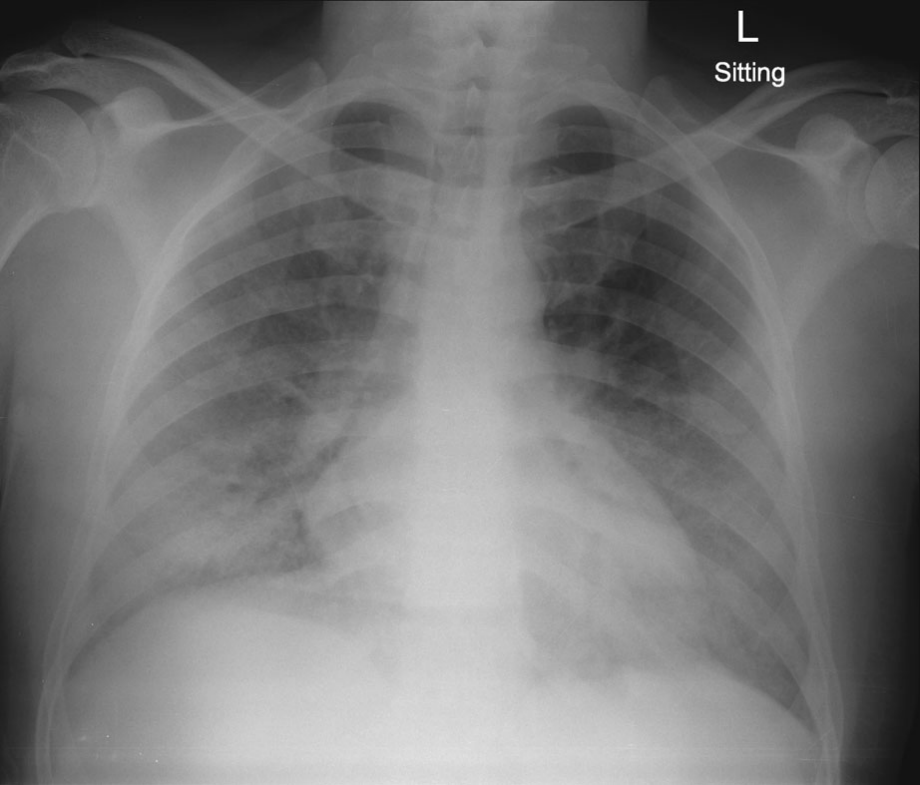
The patient's chest radiograph on admission showed bilateral, predominantly lower zone, ground‐glass opacifications.

Within 24‐h of transfer, his oxygen requirements increased and he was transferred to our intensive care unit (ICU) for high‐flow nasal cannula (HFNC) oxygen. On arrival, he was alert and haemodynamically stable with a respiratory rate of 32 and SpO_2_ of 98% on a non‐rebreather facemask. Initial arterial blood gas analysis performed while receiving high‐flow nasal oxygen at 60 L/min at a fraction of inspired oxygen (FiO_2_) of 40% showed partial pressure of oxygen (PaO_2_) of 8.7 kPa, partial pressure of carbon dioxide (PaCO_2_) of 3.9 kPa, and pH of 7.5. This confirmed type 1 respiratory failure with a PaO_2_:FiO_2_ ratio of 163. His chest radiograph (Fig. 1) showed bilateral, predominantly lower zone, ground‐glass opacifications.

His blood results showed a raised white cell count (WCC) of 15.1 × 10^9^/L, with a lymphopaenia of 0.69 × 10^9^/L, a C‐reactive protein (CRP) of 157 mg/L, a procalcitonin of 0.58 μg/L, and a raised D‐dimer level of 1.02 mg/L. The remainder of his haematology tests, as well as renal and liver function blood tests were unremarkable. Sputum GeneXpert MTB/Rif Ultra (Becton Dickinson, USA) testing as well as his urinary lipoarabinomannan (LAM) were negative. Regular cultures of blood, urine, and sputum were sent, as well as sputum for a direct fluorescent antibody test (DFAT) for PCP, serum beta‐D‐glucan, and a urinary Legionella antigen test.

**Figure 2:**
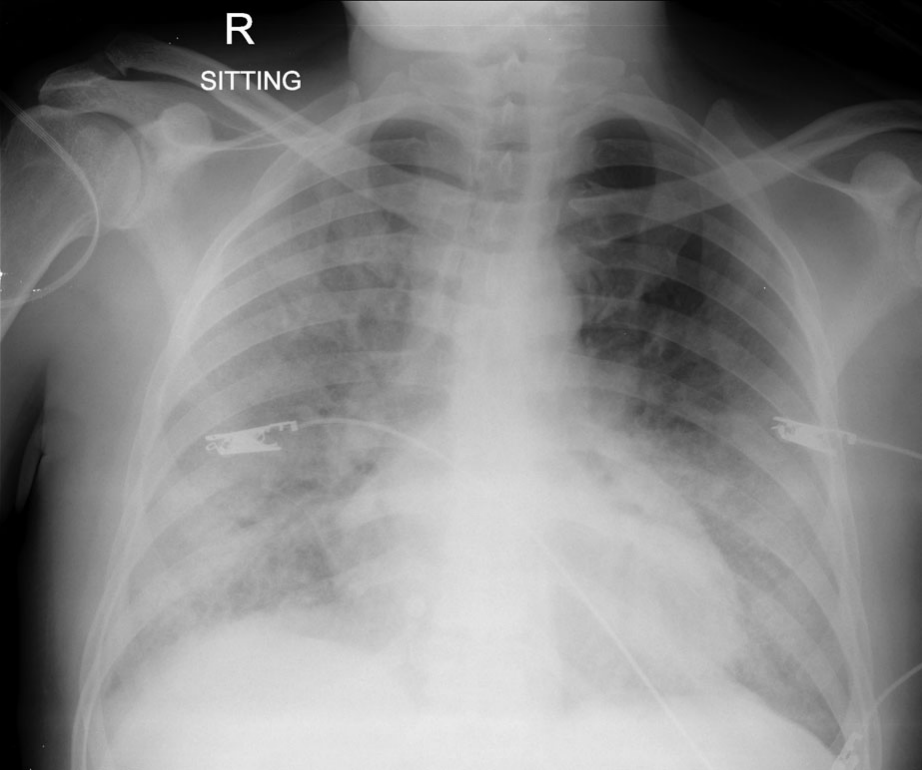
This chest radiograph showed significant worsening with consolidation involving all but the left upper zone.

A working diagnosis of COVID‐19 and/or PCP was made and he was initiated on empiric dexamethasone and therapeutic cotrimoxazole. However, on the third day of admission, a diagnosis of nosocomial pneumonia was made on the basis of persistent fever, haemodynamic instability, increasing oxygen requirements with a worsening pulmonary infiltrates on chest X‐ray (Fig. 2), and rising inflammatory markers. Empiric meropenem and fluconazole were initiated pending culture results.

PCP was confirmed on sputum DFAT and supported by a raised beta‐D‐glucan level of >500 pg/mL (normal <60). In addition, blood cultures grew *Acinetobacter baumannii* for which colistin was commenced pending sensitivities. Urine testing for Legionella species was negative.

The patient demised on day 8 of admission to ICU from progressive respiratory failure.

## Discussion

In high HIV prevalence settings, the diagnosis of PCP is frequently made presumptively using case definitions that include clinical features and a compatible chest X‐ray in an immunosuppressed individual with a CD_4_ < 200 cells/μL [3, 4, 5]. This pragmatic approach is largely driven by limited availability of bronchoscopy and the relatively poor sensitivity of DFAT on sputum [5, 6, 8]. However, the clinical overlap with COVID‐19 impacts the accuracy of the clinical case definition for PCP. Interpretation of PLWH's CD_4_ counts and assumption about immune status are additionally problematic in the setting of SARS‐CoV‐2 infection. Furthermore, if SARS‐CoV‐2 becomes persistent in theses populations after the current pandemic dissipates, purely syndromic diagnoses for PCP in the future may miss COVID‐19 if testing is not performed.

Identifying co‐infection is additionally complicated by the poor sensitivity of both the nasopharyngeal PCR for COVID‐19 and the sputum DFAT for PCP, with sensitivities of 63% [9] and 55% [7, 8], respectively. Thus, both tests may yield significant false‐negative rates, providing false reassurance to clinicians and potentially putting patients at risk. CRP, being a sensitive marker of infection, is unhelpful in differentiating COVID‐19 from PCP, being frequently elevated and corresponding to disease severity in both [4, 10, 11].

Subtle clues differentiating PCP from COVID‐19 on computed tomography (CT) imaging may be a more central distribution of ground glass in PCP with peripheral sparing in 41%, with some describing a predilection in the upper lobes, and cysts in up to one‐third of cases [12]. In COVID‐19, the ground‐glass opacifications have a peripheral predominance in 76% of cases and cysts are uncommon [13].

The individual contribution of each pathogen to disease severity in this patient is unclear, and the extent to which dual COVID‐19 and PCP interact is unknown. Histological and immunological testing would be required to answer these questions and, in future cases, should be pursued. In all likelihood, his death was ultimately caused by acquired nosocomial sepsis.

Clinicians working in populations with high HIV burdens need to consider PCP as a differential in a patient with COVID‐19 pneumonia and consider empiric treatment while awaiting a definitive diagnosis. Pitfalls remain around imaging and definitive diagnostic testing.

### Disclosure Statement

Appropriate written informed consent was obtained for publication of this case report and accompanying images.
